# Supplementary material for: Are carbon emissions trading and green financial instruments synergistic? -Comprehensive quantitative research based on content analysis
Source: PLoS One. 2024 Mar 7;19(3):e0298601. doi: 10.1371/journal.pone.0298601 (PMC10919601; doi:10.1371/journal.pone.0298601)
Supplement: S1 Data — (ZIP) [file pone.0298601.s001.zip › Data/地级市Z_F工作报告.zip!/║┌┴·╜¡/╦τ╗»╩╨/└┤╫╘╣1⁄2╓┌║┼í╛┬φ┐╦╩2╛▌═°í┐_2016.docx]

各位代表:

我代表市人民政府，向大会报告工作，请予审议，并请各位市政协委员提出意见。

一、“十二五”时期及2015年工作回顾

“十二五”时期，按照市委确定的战略思路目标，深入解放思想，坚持改革推动、开放带动、创新驱动，扩投资、兴产业、强功能、优环境、聚人气，圆满完成各项任务目标。这五年，是全市经济质量效益不断提升的五年，是城乡面貌变化最大的五年，是社会各项事业发展最快的五年，是人民群众得到实惠最多的五年，是各级干部、广大群众努力拼搏、团结奋进的五年。

——经济总量实现跃升晋级。GDP突破千亿大关，总量上升到全省第四位。累计固定资产投资3007亿元，是“十一五”的3.4倍。公共财政预算收入56.2亿元，是2010年的近2倍。规模以上工业增加值年均增长16.4%，是2010年2.1倍。社会消费品零售总额年均增长15.3%，是2010年的2倍。城乡存款余额1130亿元，是2010年的2倍。

——产业项目建设取得重大突破。“十二五”时期是产业项目大发展时期。11个产业园区规划面积346平方公里，建成区93平方公里，入驻企业1036户。建成1个国家级、5个省级开发区。产业园区成为绥化的重要增长极。招商引资签约产业项目4409个，象屿、大唐、恒大、中粮等一批大集团、大项目落户绥化。累计开工建设产业项目2416个，投产1585个。形成了全党动员，人大政协合力，全市上下全民招商的浓厚氛围。规模以上工业企业366户，是2010年的2.3倍。高新技术产业产值、增加值年均增长20%。产业项目建设总体水平位居全省前列。

——现代农业建设迈上新台阶。两大平原现代农业综合配套改革深入推进，新型经营主体发展到4.4万户，规模经营比重达到63%。粮食产量实现“十二连增”，10个县市区全部跻身全国产粮大县行列，被确定为全国整市(地)整建制农业高产创建示范市、粮食生产先进市、国家级现代农业示范区和中国绿色蔬菜生产基地北菜南运核心区。建成现代农业园区606个，累计获国家奖励资金超过40亿元。水稻智能浸种催芽能力覆盖面积90%以上。建成现代农机合作社253个，综合机械化率达到94.9%。农业科技贡献率提高4个百分点。累计完成造林绿化109万亩，农田水利设施得到明显加强。市级以上产业化龙头企业发展到157户，加工转化能力1300万吨，玉米、亚麻、生猪加工能力居全省前列，农产品精深加工率达到38.5%。在北京、上海等大中城市建设绿色食品品牌旗舰店、连锁店200多家。畜牧业增加值增长1.05倍。绿色食品生产基地面积和“三品一标”认证总量均居全省前列。现代农业建设实现重大突破。

——城乡面貌发生巨大变化。全市常住人口城镇化率提高6.4个百分点，九县市城区面积扩大21.2平方公里。投入66亿元建设“三供三治”项目60个，城区道路、给排水、垃圾污水处理、绿化大幅改善。新增供热面积3200万平方米，城市集中供热普及率达到85%，污水集中处理率达到70%。新建改造公园广场67个，新增城市绿地1190万平方米，人均公园绿地面积达到8.4平方米。完成交通投资59.8亿元，路网总里程2万公里，伊绥、绥北高速建成，城乡路网实现全覆盖连接。26个乡镇列入全国重点小城镇，市本级、庆安、北林部分乡镇实现垃圾焚烧发电。几年来的变化，楼高了、路宽了、灯亮了、地绿了、城美了。

——民生水平持续提高。民生投入占财政支出比重64.1%。城乡常住人口人均可支配收入首次突破2万元和1万元。改善办学条件投入37亿元，新建学校、幼儿园220所，改扩建学校、幼儿园207所；建设标准化义务学校218所。招聘本科以上学历教师3516名。新建县级医院11所、乡镇卫生院65所、村卫生室480个，招聘高水平医护人员1616名。医疗机构总床位数增长21.3%，业务用房面积增长137.7%。全市专利申请量、授权量比“十一五”增长一倍。新农合在全省率先实行大病保险。城镇累计新增就业32万人，失业再就业29万人，零就业家庭保持动态为零。城乡低保应保尽保，标准分别提高128.5%和191.7%。205个村、22.8万人实现脱贫。新增193万人饮水安全。累计新建保障性住房13.4万套，城市人均住房面积增加5.5平方米。农村泥草(危)房改造连续保持全省第一，住房砖瓦化率提高15个百分点。图书馆、文化馆、博物馆、纪念馆等大型文化场馆总面积近100万平方米,比2010年增长73.8%。

——市本级经济社会快速发展。公共财政预算收入8.5亿元，是2010年的2.1倍。规模以上工业增加值6.9亿元，是2010年的1.3倍。开发区晋升为国家级经济技术开发区。城市建成区面积达到45平方公里，新增15.3平方公里，人均住房面积35.5平方米。投资5.7亿元新建改造科研路、黄河路、康庄路及城区主次干道，街路巷道全部实现硬覆盖。投资12.6亿元，开复工“三供三治”项目20个。投资4.7亿元，新改扩建人民公园、西湖公园、森林公园等14处。投资8700万元，进行城市绿化。改善办学条件资金达到3亿元，新建第七中学、第十中学、实验幼儿园、长江小学。重点院校录取率提高12.7个百分点。新改扩建妇幼保健院、人民医院，面积4.5万平方米，市直医院医疗设备投入1.5亿元。

通过“十二五”的工作实践，我们深刻地体会到，必须紧紧依靠市委的坚强领导，深入贯彻落实市委的战略，是我们做好各项工作的总纲，是我们各项事业不断取得发展进步的根本保证；必须坚定农区现代化的发展战略，坚持经营农业思想，推进经济工作项目化，厚植优势，深耕潜力，城乡统筹，四化联动，引领绥化走向更富更强；必须坚持改革创新，真抓实干，迎着困难上，滚石上山，爬坡过坎，定下的事情一抓到底，就能够推动各项事业不断迈上新台阶；必须致力改善发展民生，作为政府工作的出发点和落脚点，更加注重包容性发展和共享式发展，把富民和强市有机结合起来，切实增强广大人民群众的获得感；必须严守政治纪律和政治规矩，廉洁自律，认真践行“三严三实”要求，自觉接受人大依法监督、政协民主监督和社会各界监督，打造忠诚干净担当的政府。

刚刚过去的2015年，在市委的坚强领导下，围绕三个导向，坚持四化联动，大力推进农区现代化，经济社会保持健康发展。实现地区生产总值1274亿元，增长7%；固定资产投资697亿元，增长10%；社会消费品零售总额510亿元，增长10.3%；公共财政预算收入56.2亿元，下降8.7%。

1、产业项目和工业经济势头强劲。大力推进招商引资和产业项目建设。签约产业项目777个，合同资金1763亿元。实际利用外资2.6亿美元，增长11.3%。开复工500万元以上产业项目622个，完成投资290亿元，新开工440个，推进5亿元以上重点项目32个。新增规模以上企业35户，规模以上工业增加值实现220亿元，增长6%。非公经济增加值增长7.8%。食品、能源、石化产业比重达到73.1%，主导产业贡献率进一步提高。

2、现代农业建设步伐加快。以科技示范带、智能示范区为载体，建设互联网+示范园区161个，“三减”试验示范39个点次，新建现代农机合作社13个。土地确权全面推开。阁山水库正式开工，十六道岗水库有望列入省“十三五”规划。粮食总产269.2亿斤，绿色食品种植面积1650万亩，蔬菜等经济作物面积扩大23%。新建标准化畜牧园区、养殖场123个。肇东被确定为国家现代农业示范区改革与建设试点县，连续3年每年获得支持资金4000万元。

3、城乡建设提档升级。坚持改造老城区、辟建新城区、提升开发区，大力推进美丽乡村建设。市政基础设施建设完成投资3.3亿元，绿化投资1.5亿元。实施城市“三供三治”项目39个，完成投资12.2亿元。棚户区改造开工14534套，消化存量房60万平方米。改造泥草(危)房3.4万户，建设购买公租房2335套。启动38个重点集镇、176个示范村建设，完成投资17.9亿元。

4、现代服务业繁荣活跃。注重提高服务业经济比重和贡献率。建设9个电子商务产业园，全市电子商务交易额51亿元。寒地黑土品牌回归绥化。新增金融机构9家，存贷款余额分别增长17.1%和23.2%。20户企业在“新三板”和区域股权交易市场挂牌。旅游业收入突破10亿元。

5、民生福祉持续改善。城镇新增就业5.9万人。投入低保资金13.2亿元、扶贫资金7.4亿元。建设义务标准化学校37所。补充乡镇医护人员185名。新增养老机构28家，床位2711张。完成全民健身工程200处。投入3.8亿元，解决了57.7万人饮水安全。农村环境连片整治投入3.2亿元。圆满完成了“六五”普法规划，法治绥化、平安绥化建设有力推进，社会和谐稳定。

6、改革创新深入推进。坚持“多取消、审一次、真备案”，深化行政审批制度改革，189项市级行政审批取消或下放，34项非行政许可全面取消。编制完成权力清单、责任清单和权力运行图。公共资源配置市场化改革取得较大进展，市场化集中供热热源14处、污水处理9处。医院、普通高中全面推行绩效工资，事业单位“三权分离”改革进展顺利。市本级预算单位全部实行两级核算、三级管理。厂办大集体改革深入推进。卫生计生、食品药品、工商质监等机构改革顺利完成。

7、区域中心城市面貌日新月异。突出完善功能，提升品位。获得全国文明城市提名资格。启动建设东部生态新城。大唐热电联产和绥大高速建设进展顺利。投资8600万元，建设数字绥化、智慧绥化。大力推进三年棚改攻坚，重点实施“三供三治”工程7项，完成投资6.3亿元。新建改造公园广场11处。投资2000万元对60个老旧小区进行二次供水改造。完成了市区20个小区物业综合整治试点，理顺了小区物业管理体制。十个方面民生实事全部兑现。被评为“全国六星级慈善城市”。经济技术开发区新续建产业项目31个,完成投资12.6亿元。

8、政府自身建设不断加强。深入开展“三严三实”专题教育，大力整治不作为、乱作为、慢作为。完善决策程序机制和法律顾问制度，政府决策更加民主，更加符合法律规定。加强依法行政，优化服务，提高效率，行政效能不断提高。政府投资严格招投标、财政评审、决算审计。清理规范中介机构，加强企业投诉中心建设。市本级开展清理政府部门非办公类资产，“三公”经费下降10%。从严落实“一岗双责”，把政治纪律政治规矩挺在前面，政府系统党风廉政建设取得明显成效。

公共节能、民族宗教、外事侨务、食品药品安全、国防动员、妇女儿童、统计监测、农业开发、公积金、残疾人、防灾减灾、史志档案、气象人防、老龄双拥等各项工作都取得新成绩。

各位代表！经过五年来，特别是2015年的不懈努力，全市经济社会发展站在了新的起点，为“十三五”精彩开局和2020年全面建成小康社会、全面脱贫奠定了坚实基础。发展令人振奋，成绩来之不易！这是省委、省政府和市委正确领导的结果，是社会各界积极献计出力的结果，是全市人民共同奋斗的结果。在此，我代表市人民政府，向积极参与、全力支持绥化建设发展的广大干部群众、各界人士、解放军、武警官兵，表示衷心的感谢，并致以崇高的敬意！

在总结成绩的同时，我们清醒地认识到，全市经济社会发展还面临很多压力和挑战，政府工作还存在不少困难和差距。

一是经济增长和财政增收的动力不足，产业结构不优，市场化程度不高，发展环境还需大力改善；二是城市基础建设还存在差距，功能不够完善，品位和形象还有待提高；三是科技实力和创新能力不强，还缺少核心竞争力；四是民生快速改善和农村贫困人口脱贫路径不宽；五是信访维稳、安全生产压力较大；六是一些政府部门专业化程度与工作要求还有一定差距。特别是2015年由于经济下行等因素的冲击，公共财政预算收入出现负增长。对此，我们将高度重视，认真解决。

二、“十三五”时期经济社会发展主要目标

“十三五”时期，党中央、国务院、省委省政府的发展要求已经明确，市委三届八次全会做出科学部署。根据市委《关于制定绥化市国民经济和社会发展第十三个五年规划建议》，市政府深入研究，广泛听取各方意见，制定了《绥化市国民经济和社会发展第十三个五年规划纲要(草案)》，提请大会审议。推进农区现代化的发展战略，符合上级精神，符合绥化实际，符合广大干部愿望和人民群众根本利益。政府工作要按照市委部署，全面贯彻党的十八大和中央、省委省政府、市委一系列决策部署，深入落实“四个全面”战略布局，牢固树立创新、协调、绿色、开放、共享发展理念，实施好省委省政府“五大规划”，融入龙江陆海丝绸之路经济带，落实“十大产业”建设，强化“一个主基调”，把握“两个阶段性特征”，围绕“三个导向”，坚持“四化联动”，加速农区现代化进程，统筹推进经济、政治、文化、社会、生态建设，奋力夺取全面建成小康社会决战决胜。

主要预期目标是：地区生产总值年均增长7.5%；固定资产投资年均增长10%；社会消费品零售总额年均增长10%；公共财政预算收入年均增长8%；规模以上工业增加值年均增长8%；城乡居民人均可支配收入年均增长8%。到2020年，与全国全省一道全面建成小康社会，一道实现全面脱贫目标。

——从促进结构调整、全面深化改革入手，加快形成促进创新的体制框架，解决发展动力问题，推进创新发展实现新突破。努力破解结构性和体制性制约，增强发展动能。推进一产由大到强，提高二产牵动力，增强三产贡献率。做大做强绿色食品产业，发挥资源优势，扩充经济总量；大力发展新能源新材料战略性新兴产业，强化发展动力；优化粮经饲比例，调整到7:2:1，为工业经济发展提供优质原料；大力发展畜牧业，占农业比重达到50%以上，推进“主辅双强”。进一步提高园区集聚力和产业集中度，形成具有比较优势的加工板块和产业集群。三次产业比达到35.5：28：36.5。

——从统筹城乡、四化联动入手，加快补齐“短板”，解决发展均衡性问题，推进协调发展提升新水平。推进“四化联动”，统筹区域、城乡、经济社会、人与自然协调发展，实现城乡要素平等交换、合理配置，有效发挥农业现代化的基础作用、工业化的拉动作用、城镇化的升级作用、信息化的激活作用。突出加快发展壮大市本级，提升城市能级，增强中心城市对要素、项目、人才的集聚吸引，提高辐射能力；强化肇东、安达两个十强县的示范带动，促进六个贫困县后发追赶，实现区域、产业、软硬实力协调发展。

——从巩固提升整体生态优势入手，走好经济技术和社会能力双提升的绿色发展道路，提高生产生活品质，推进绿色发展取得新成效。叫响“绿色绥化”主题，强化“绿色增长”理念。转变生产生活方式，推动集约循环可持续，生态与发展融合互促。万元GDP能耗年均降低3.2%，森林覆盖率达到12.7%，城市人均公园绿地达到12平方米。秸秆综合利用率达到90%以上，循环经济比重进一步提高。化肥、农药、除草剂施用量分别下降10%、20%和20%。坚决治理污水、黄标车和不达标锅炉，全面禁烧劣质煤。

——从全方位融入龙江陆海丝绸之路经济带入手，充分利用国际国内两种资源两个市场，拓展发展空间，推进开放发展迈出新步伐。加强以俄、日、韩为主的国际市场开拓，壮大对外经贸群体，外贸进出口额年均增长7%以上。加强交通、物流、信息三大建设，提高基础设施互联互通能力，加快打造营口陆港等一批重点项目，推进对外贸易便利化。落靠“五个盯住”(盯住哈尔滨新区建设、盯住哈尔滨人才、盯住哈欧班列、盯住哈尔滨产业转移、盯住哈尔滨消费市场)和“四互四共”(交通互联共建、信息互通共享、产业互补共兴、合作互惠共赢)，努力打造中蒙俄经济走廊龙江陆海丝绸之路经济带上的重要节点城市。

——从根本上改善民生福祉入手，作出更有效的制度安排，维护社会公平正义，推进共享发展进入新层次。坚持普惠性、保基本、均等化方向，大力推进脱贫攻坚、社会保障、社会治理三大工程，推进教育、科技、文化、卫生、体育等公共服务均等化。完善社会保障体系，深入实施“七五”普法规划，建设安全、公平、关爱的社会环境。人人参与、人人尽力、人人享有，让贫困人口过上“好日子”、真正“奔小康”，达到“两不愁三保障”(不愁吃、不愁穿，义务教育、基本医疗、住房安全有保障)，创造安居乐业的美好生活。

三、2016年政府主要工作任务

2016年是“十三五”和全面建成小康社会决战决胜开局之年，做好今年的工作尤为重要。要认真贯彻落实省委、省政府五大发展规划和龙江陆海丝绸之路经济带战略，深入抓好市委提出的“五个着力推进”、“七项重点工作”、“七个坚持”，提高落实力，增强执行力。

主要预期目标是：GDP增长7-7.5%；固定资产投资增长10%；社会消费品零售总额增长10%；城乡居民收入增长8%；规模以上工业增加值增长8%；公共财政预算收入增长6%。

重点做好十个方面工作。

（一）坚定经营农业理念，加快现代农业发展

坚持巩固完善提高，加快转变发展方式，调整优化结构，推进三产融合，使现代农业的优势和潜能得到充分释放。

调整优化结构。以提高粮食综合生产能力，保障国家粮食安全为核心，大力发展优质高效农业、畜牧业、食品工业。顺应市场需求，减玉米、扩经济作物和饲料作物。围绕熟悉的技术调整，促进增产增收。积极推进三产融合，大力发展农产品精深加工，扩大专用品种面积。促进农畜结合，发展青储玉米和饲草饲料。大力发展蔬菜产业，种植面积增长15%以上。抓住市场需求，立足本地市场和能够辐射到的市场，把生态绿色资源优势变成农产品品质优势，实现既要种得好更要销得好。

发展绿色农业。以2000公里科技示范带为载体，以11个智慧农业园区为核心，建设互联网+绿色农业示范园区600个。加强种子繁育基地和加工体系建设。推动耕作制度的制定和实施，继续扩大“三减”试验示范，推广面积500万亩，化肥、农药施用量分别减少0.4公斤和0.07公斤。强化耕地保护，测土配方施肥面积1500万亩。全面推广绿色投入、绿色防控和水稻测深施肥技术，深松整地800万亩，秸秆还田达到三分之一。

加强设施建设。坚持用好天降水、过境水、再生水，科学开采地下水，构建旱能灌、涝能排的水利设施体系。发挥好农机合作社的作用，与农民合作社有机嫁接，推进合作社由生产主体向全方位经营主体转变，新建农机合作社20个。推进科技创新，打造完善的服务基层平台。深化与省农科院合作，加快科技成果转化。继续推进亿亩生态高产标准农田建设。

加快畜牧业发展。大力发展草食畜牧业，稳定发展耗粮型畜牧业，重点建设优质肉牛基地、绿色奶源基地、寒地黑土生猪基地、优质大鹅基地。在草食畜牧业发展上力争实现大的突破，争取建设7个万头肉牛养殖场，10个10万只大鹅养殖场。畜牧业增加值增长7%，拉动农民收入增长4%，加快实现“主辅双强”。

推动农民创业。坚持思想发动、典型示范，“帮、学、干”结合，鼓励支持农民从事能够增加收入、把生产出来的农产品以高于政府收购价卖给市场的种植、养殖、销售、加工等多种产业化创业活动。以创业带动走出土地进入城镇稳定就业，促进农民多元增收，农民收入增速要高于经济发展速度。

（二）突出产业项目建设，提升工业经济整体实力

通过巩固、招商、改造、升级，进一步扩大增量，提高投资有效性，培育壮大立县、立市支柱产业，提升产业竞争力。

向巩固要增量。巩固已经上马、落地、投产的产业项目，这是全市经济发展的基石。巩固现有企业，巩固良好环境，巩固外部影响力，巩固管理经验，巩固已经占有的市场。在建续建项目年内投产率达到50%，投产项目80%以上达产达效，60%企业有新产品、新市场。

向招商要增量。继续坚持全党、全民、全社会招商，重点盯住与我市契合度高的500强大企业、大项目，落实责任部门，长期跟踪。全市引进产业项目要达到600个以上。新上产业项目数量和完成投资额提高20%以上。注重引进科研成果、管理经验、发展模式、专业人才，提高核心竞争力。

向改造要增量。盘活存量，延长产业链条，实现裂变发展。推进技术改造、产品开发、商业模式创新。加快盘活僵尸企业，推动制造业绿色改造，兼并重组一批、强化管理一批、淘汰落后一批，提高资源利用率。实施技术改造20项，停产半停产企业同比减少20%。

向升级要增量。突出150户重点企业，提高竞争实力，加大成长型企业培育力度。强化市场营销，产值10亿元以上企业发展到15户，利税亿元以上企业发展到27户。大力推进律师事务所、会计师事务所、资产评估所和金融机构进园区。发展规模较大科技型企业15户。市本级和兰西两个科技企业孵化器建成投入使用。

（三）推进转型升级，提高服务业贡献能力

扩大需求和有效供给两侧发力，多角度、多领域推动服务业发展，提高服务业发展层次、经济比重和贡献份额。

积极主动融入龙江丝路带。围绕全省“一核四带一环”产业布局，谋划建设进出口加工产业园、国际物流园，建设肇东大庄园、望奎双汇北大荒、青冈赛美葵花、绥棱林产品等一批外贸进出口龙头企业和基地。借助哈欧班列，谋划一批农业、绿色食品、工艺品等合作项目。加快营口陆港建设，积极争取设立保税仓，提高通关便利化。大力发展对俄跨境电子商务。进出口贸易总额增长5%，实际利用外资增长7%。

提高现代服务业发展水平。积极引进建设具有拉动经济、提升城市品位的大型商贸综合体。全年引进现代服务业项目40个以上。加快现代物流业发展，大力发展冷链物流。推动市本级和各县市物流园区升级，加快第三方物流发展，打造区域物流中心节点，提升城乡物流配送水平。

围绕消费热点打造新经济。大力发展农业观光、养生度假、民族文化等特色旅游，打造具有区域竞争力的特色旅游产品。发展大健康产业，深度发展保健用品、营养食品、休闲健身产品，加快投资3亿元的健康生物科技产业园建设。发展养老产业，每个县至少扶持1个100张床位以上的“医养结合”型养老机构。

创新金融服务业。打造优良的金融生态环境，大力引进各类金融机构，拓展融资渠道，鼓励企业直接融资，努力提高存贷比。争取10户以上企业在新三板、战略新兴板挂牌。创新农村金融产品，全面开展农村土地承包经营权、农民住房财产权抵押试点，扩大林权抵押贷款规模。提高农业保险服务覆盖面。

完善“互联网+”平台建设。深入实施“互联网+”行动计划，开展电子商务示范县、示范园区、示范企业和生产基地建设，全面提高基础保障能力。鼓励支持绿色食品企业、外贸企业开展跨境贸易。

进一步做大寒地黑土区域公用品牌。叫响“寒地黑土、绥化物产、健康营养”品牌内涵。强化品牌集中度，加速与地方特色品牌、地理标志产品有机融合。集中打造绥化“绿都”、“硒都”形象。扩大“北菜南运”寒地黑土品牌使用面。

（四）建城管城兴城并重，提高城乡发展水平

尊重城市发展规律，统筹空间规模产业，统筹规划建设管理，统筹改革科技文化，统筹生产生活生态，统筹政府社会市民，打造具有农区特色的新型城镇化体系。努力实现城市让生活更美好、环境让百姓更宜居、发展让人们更幸福、文化让绥化更和谐。

加快市本级中心城市建设。突出提升城市品位和能级。践行地下管廊、海绵城市建设理念，提升城市通透性和微循环能力。坚持长远规划，突出城市功能定位、文化特色，满足80万人口整体功能的需求。加强城市设计，空间立体性、平面协调性、风貌整体性、文脉延续性统筹协调，公益布局科学合理，生态功能完善发展，力争50年不落后，100年是个景。重点加快东部生态新城建设，以火车站及站前广场改造为核心，以城南河和东湖为水系和景观，以北林路和太平街为重要节点。今年重点推进火车站改造、41厂棚户区改造、城南河治理、绥巴路改造等8项重点工程，抓好绥南、绥西、绥兰、绥望、绥巴出入城口改造升级。完善中心城市功能，加快补上博物馆、文化艺术中心、综合体育场等文体设施短板，提升教育、卫生整体水平和层次。加强城市精细化管理，推进数字化平台功能整合，建设综合性城市管理数据库。充分发挥社区在社会治理创新中的作用，提高市民文明素质，推进城市共治共管、共建共享，培育良好的城市文化。

加快县城建设。突出抓好县城建设，强化功能，建成承载人口、吸纳就业、改善生活质量的重要承接地。坚持规划建城、设计建楼，建设新区、改造老区、完善小区，提升功能，增加公益。高标准、高质量、高效能推进路、水、电、热、气、通讯、公交建设，完善教育、卫生、文化、体育等公共服务。开复工“三供三治”工程40项。坚持政府主导、市场运作，以需定建，以质促销，利用棚改政策和信贷支持，加快去库存。完善政策吸引农业人口进城。

加快小城镇建设。小城镇是接收农村人口提高城镇化率、缩小城乡差别的重要辅助。突出特色，打造城乡统筹的重要节点。坚持注重计划性，科学规划设计，独具特色、自然纯朴。坚持先基础、公益，后一般，推进成小区、成规模开发、改造、建设。加强供热、供水、排水、物业等配套建设，做到有质量、有个性。利用当地资源优势，发展产业，增加就业，形成城镇化重要支撑。

加快美丽乡村建设。推动建设内容提档升级，坚持与扶贫结合，与泥草(危)房改造结合，与产业发展结合，搬得进、住得好、促增收。尊重原生态自然环境，尊重农民意愿，尊重历史文化。抓好治理脏乱，植树绿化。打造一批具有示范引领作用的典型重点乡镇、示范村，建设重点乡镇42个、示范村达标村330个。

（五）推进呼兰河流域综合开发，厚植发展新动能

呼兰河是绥化的母亲河，全长525公里，流经我市境内345公里，占全长的65.5%。按照市委三届八次全委(扩大)会议精神，推进呼兰河流域综合开发，打造黑龙江两大平原现代农业综合配套改革试验创新示范区。主要分两大区域。一是核心区。主河道两侧10公里之内，10条主要一级支流两侧5公里之内，其他支流两侧2公里之内。二是辐射带动区。安达、肇东有河流的区域及其他8个县(市、区)核心区以外的范围。重点做好控水、节水、净水、兴水文章，建设围水项目，做强围水产业，发展围水经济。在强化基础和配套设施的基础上，兴农业、上畜牧、办加工，使整个流域农业强起来、农民富起来、农村美起来。今年重点抓好六项工作。

编制综合性开发规划。市里完成综合开发和水利专项开发规划编制工作，各县(市、区)制定相应具体规划，加快推进。

全力推进重点项目。集中力量抓好阁山水库项目建设，十六道岗水库力争纳入国家“十三五”盘子。规划好引通(通肯河)入红(东方红水库)、引拉(拉林河)入柳(柳河水库)工程。抓好兰西河口水电站橡胶坝水毁修复、各支流河道清淤和旅游开发、北林幸福水库除险加固等50个重点项目推进。每个县(市、区)启动2-3个重点项目。相关部门提出具体项目列入整体规划。

注重生态修复保护。恢复植被，还林、还草、还湿，还原于原生态优于原生态，建设美丽家园。

发展滨水经济。搞好水系联通，注重三产融合，促进滨水城镇村一体化发展。搞好山、水、林、田、路综合治理。

加强污水治理。对向呼兰河排放的水体进行严格管控，对沿河排污口进行在线监控，全面提高呼兰河水质。

加大宣传力度。营造综合开发的良好舆论氛围，积极争取国家和省各有关部门及社会各界更多的支持。

（六）改善发展民生，提高社会事业发展水平

加大民生投入保障力度，推进各项社会事业全面发展。

提高社会保障能力。积极推进养老保险扩面征缴，新增参保人员2万人，基本完成医疗保险市级统筹。完善商业保险机构参与新农合经办服务。加快发展养老服务业，积极推进“医养结合”，鼓励支持民办养老机构发展，新增床位2000张。做好工伤、失业、生育保险工作。

努力扩大就业再就业。深入开展“双创”，做好就业困难人员和高校毕业生就业工作。做好厂办大集体改革社会保险接续工作。规范用工，加大劳动保障监察力度。力争完成城镇新增就业4.6万人。

提高社会救助水平。推动医疗救助“一站式”结算，深入开展“救急难”工作。严格落实救灾资金分级负担的保障机制。做好救助管理和儿童收养工作。积极做好双拥安置优抚工作。支持发展公益慈善事业，推进全国星级慈善城市建设。

完善公共服务体系。推进义务教育均衡发展，加快发展学前教育和职业教育，建设义务教育标准化学校30所。新改扩建乡镇卫生院15所、村卫生室170个。新建健身工程100处以上。完善城乡公共文化基础设施，推进文化场馆达标建设，加快市场化运作文体设施项目。建立信息化服务平台示范社区30个，建设700个数字新闻服务站。

推进重大项目建设。抓好保障房、水利、交通等“一号工程”，提高民生保障水平。推进保障性安居工程。发挥政府主导作用，高标准完成三年棚改任务。改造棚户区880万平方米，去库存290万平方米。改造农村泥草(危)房3万户。推进重大水利工程。确保重点水利工程建设质量和工期，全面落实千亿斤粮食产能工程。推进重大交通工程。加快绥大高速公路、哈绥铁路电气化改造、中俄天然气管道支线工程建设。积极谋划绥化机场、哈绥城际铁路等重点项目。抓好哈黑公路兰青明段升级改造。新建改造农村公路1000公里。

（七）坚持精准发力，打好打赢脱贫攻坚战

落实落靠“扶持谁、谁来扶、怎么扶、如何退”，突出精准扶贫、精准脱贫，增强针对性，提高实效性。

明确任务目标。2019年全部完成脱贫攻坚任务。2017年绥棱实现摘帽，2018年青冈、望奎、兰西、海伦、明水实现摘帽。今年完成100个重点村整村推进、12万贫困人口脱贫任务。

解决突出问题。重点解决贫困县中真正的贫困人口和非贫困县中贫困人口的脱贫问题，通过产业、创业、教育、医疗等措施推进精准扶贫。严格把握“两不愁三保障”标准，在提高收入的基础上，加大泥草(危)房改造力度，提高基本医疗保障和义务教育均衡发展水平。

创新脱贫措施。深入落实“五扶一抓”。扶脱贫志气，教育引导贫困村和贫困户强化自力更生、自我发展意识；扶职业技能，开展劳动技能培训，提高困难群众就业创业能力；扶发展条件，完善贫困村屯基础设施、公共设施建设；扶增收产业，发展富民产业，提高贫困户参与度；扶困难群体，增强社会保障能力，加快贫困线、低保线“两线合一”。狠抓责任落实，落靠主体责任，推进齐抓共管。加大抓典型、推模式力度。

（八）全面深化改革，释放发展活力

加大重点领域和关键环节改革力度，增动力、添活力。

推进供给侧结构性改革。坚持三个导向，推进“三去一降一补”，提高投资有效性，通过新供给，释放新消费，提高经济发展质量效益。加快技术改造、产品转型、市场开发、资产重组，盘活“僵尸企业”、半截项目。打通供需通道，棚户区改造与购买消化存量房有机结合去库存。推进金融创新，引导资金更好地进入实体经济。开展降低实体经济企业成本行动，降低交易成本、物流成本、税费成本，节本增效。补科技研发、产业链高端、基础设施建设、改善群众物质文化生活的短板。

深化现代农业综合配套改革。年底完成土地确权工作。新建新型经营主体3000个，增长7%。建设市级以上规范社200个。有计划有目的推进村级组织与新型经济组织有机结合，使农业生产关系更加适应农业生产力发展的要求。完善农业产业体系，打造有利于三产融合的环境和条件，实现全产业链增值。完善营销体系，加快线上线下有机结合。拓宽融资渠道，进一步规范工商资本进入农业领域。健全社会服务，抓好农业生产资料供应，全面推动优质化、规范化。深化供销合作社综合改革。

深化公共资源配置市场化改革。推进事业单位三权分离改革。将闲置的、可市场化的公共资源纳入市场化配置改革范围，积极引导社会资本参与城市公共基础设施建设。调整优化投融资体制机制，经营好发展好城投公司，重点领域建设项目开展股权和债权融资。

深化行政体制改革。简政放权、优化服务，认真执行不断完善"两单一图"制度，建设好网上审批平台。分类推进事业单位改革，稳妥有序推进机关事业单位养老保险制度改革。推进强乡扩权试点。

深化营商环境改革。进一步清理规范行政服务、中介服务、市场服务，健全完善涉企收费目录清单制度。落实降低社会保险费政策，创造良好诚信环境，降低企业制度性交易成本。严查破坏环境人和事，让企业感受到发展环境的明显变化。

（九）推进社会治理创新，积极构建和谐社会

落实精细化治理要求，加强“和谐绥化”建设，为经济社会发展和群众生产生活营造良好环境。

保障公共安全。深入抓好生产安全、食品安全、交通安全、治安安全。强化责任落实，加强日常监管，实施严管严罚。做好防灾减灾工作。推进质量兴市，提高“质量绥化”建设水平。健全城市应急体系，提升应对突发事件的能力。

维护社会公平。推进落实征收公平、机会公平、司法公平、规则公平，完善救济渠道。运用多种手段化解社会矛盾纠纷，切实维护群众合法权益。 加强社会关爱。坚持群众利益无小事。关注百姓诉求，关爱困难群体，做到群众的诉求有人听，群众的事情有人管，群众的困难有人帮。抓好特殊困难群体救助，认真落实加快推进残疾人小康进程实施意见。

保持社会稳定。创新社会治理，加强重大决策社会风险稳定评估，加强立体化社会治安防控体系建设。抓好网信安全，提高档案信息化水平。细化落实矛盾纠纷化解机制，做好信访工作。

巩固生态优势。打好环境整治百日攻坚战。深入开展大气污染防治，推进劣质煤锅炉改造，淘汰小锅炉和黄标车及老旧机动车，大力推进秸杆综合利用。加快推进水、土壤污染防治。

创建文明城市。积极争创全国文明城市，深入开展群众性精神文明创建活动，加强社会公德、职业道德、家庭美德、个人品德建设，推动社会风气优化、文明习惯养成、传统文化回归和正能量传播。深入开展民族团结进步创建活动。

（十）加快市本级经济发展，办好民生实事

突出开发区的火车头作用，壮大升级商贸服务业，提高民生保障水平，增强辐射带动能力。

增强经济发展实力。推进开发区加速发展。打造成全市具有引领作用的园区，名副其实的国家级开发区。加强主导产业培育，提高产业集中度和竞争力。新上5000万元以上产业项目15个。盘活14户停产半停产企业。创业大厦及绿色国际物流园、科技孵化园、大学生创业园、电子商务园、寒地黑土产业园上半年投入使用。整合规范物流产业发展，推动市区汽配企业入驻汽配城。投资2500万元，加强园区道路、亮化等基础设施建设。开发区主营业务收入增长10%以上，财政收入增长10%以上。提高市区商贸服务业整体水平。建设具有中心城市影响力的商贸综合体，培育各类专业批发市场，打造区域性商贸中心。支持面向生产经营的信息咨询、市场分析、融资担保等新兴服务业发展。立足现有基础和自身优势，统筹社会资源、自然资源、行政资源，积极探索实施各类市场化开发运营模式，打造城市自身经济圈，增加开发企业、经营业户、城市居民和财政收入。改造升级传统服务业，创新商业模式，加强休闲娱乐、家政养老、中介快递、社区功能等便民惠民服务。释放激活各类经营要素，培育成市本级经济的生成点、增长点和支撑点。

重点抓好十个方面民生实事：

一是加强保障性住房建设。力争完成27个棚户区，103万平方米改造任务。改造老旧小区80万平方米。对低收入家庭发放住房租赁补贴。

二是进一步改善人居环境。以PPP模式投资3亿元建设供热新管网、投资1.8亿元建设北郊污水处理工程。投资2000万元改造升级康庄路绿化工程。投资1500万元建设4处广场绿地。新建路灯1300基。清洗给水管网25公里。燃气入户3万户。建设"96333"电梯应急处置平台。建设数字绥化、智慧城市二期。

三是加强社区建设。调整完善社区布局，增加居民活动场所，丰富活动设施。改善社区办公条件，提高工资标准。

四是更加关心弱势群体。对市儿童福利院儿童实行免费体检和重大疾病免费治疗。对低收入贫困户给予取暖费补助。对生活困难居民进行价格救助。

五是大力发展教育事业。投资6500万元建设公办幼儿园、黄河小学、一中电教楼，改造北林两所学校操场。

六是推进文体惠民。投入1900万元，对9处广场进行改造升级。新建全民健身工程100处。投资2亿元，建设西湖游乐园。在五中西侧启动建设15000平方米市民健身综合馆。

七是化解看病难、看病贵。完成3万平方米市儿童医院主体建设。市第一医院投资2000万元购置派特CT。扩建北林精神病院。

八是推进养老事业发展。市场化投资8000万元，新增养老床位1000张。建设城市养护中心和宝山镇服务中心两个医养结合机构。

九是建设便民市场。新建育才路市场和花园街农贸市场，改造升级北小五路市场。

十是改善公共交通条件。提高公共交通服务水平。投资7000万元，在西湖公园北侧建设停车场一处。

四、切实加强政府自身建设

在全面从严治党新常态、经济发展新常态下，完成市委战略部署和各项任务目标，加强政府自身建设尤为重要。必须牢记使命，毫不懈怠，勇于担当，勤勉尽责，建设职能科学、权责法定、执法严明、公开公正、廉洁高效、守法诚信的法治政府。

一是树立与时俱进的思想理念。牢固树立创新、协调、绿色、开放、共享的发展理念，转变不适应市场经济的思想观念。切实增强提高市场化程度所必需的市场经济意识、开放意识、金融意识、创新意识，切实增强以法治、制度、规则为核心的发展环境意识。

二是着力打造学习型政府。政府各部门要系统学习与自身紧密结合的专业知识。结合重点工作，加强对政府工作人员的培训，强化专业思维、专业素养、专业方法。系统学习新思想、新理论、新观念，高度关注和适应新一轮科技革命和产业变革，推动新技术、新产业、新业态、新商业模式带动经济发展。

三是加强法治政府建设。依法设定权力、行使权力、制约权力、监督权力。坚决做到法无授权不可为，法定职责必须为。有权必有责、用权受监督、失职要问责、违法要追究。推进行政决策科学化、民主化、法治化。强化政治纪律政治规矩，“三重一大”事项向市委报告，重要决策广泛征求意见。自觉接受党内监督、人大依法监督、政协民主监督和社会广泛监督。带头加强诚信建设。

四是提高政府执行力。认真践行“三严三实”要求，“有思想、有激情、有韧劲”，履职尽责，奋发有为。对阻碍发展问题要勇于面对，敢于触碰实质性矛盾，切实纠正不作为、乱作为、慢作为，营造干事创业、风清气正的工作氛围。进一步改进文风会风，大兴调查研究之风。切实增强各级政府、各级行政公务人员攻坚克难能力和解决复杂问题能力。

五是坚持为民务实清廉。严格执行落实准则和条例，强化“一岗双责”，切实加强政府系统党风廉政建设，坚决反对“四风”，强化审计监督、行政问责，对腐败问题坚决做到零容忍。健全完善政府议事规则，严格程序，落实制度，巩固完善以制度管人、管事、管权的长效机制。

各位代表！承接十二五辉煌，开创十三五伟业，是时代赋予我们的光荣使命。新的征程已经开启，任务艰巨、责任重大。让我们在市委的坚强领导下，戮力同心、奋发有为，继往开来、乘势而上，为加速农区现代化进程，全面建成小康社会而努力奋斗！
